# Improving Survival of Juvenile Scalloped Spiny Lobster (*Panulirus homarus*) and Crucifix Crab (*Charybdis feriatus*) Using Shelter and Live Prey

**DOI:** 10.3390/ani11020370

**Published:** 2021-02-02

**Authors:** Chia-Huan Ma, Po-Yu Huang, Yung-Cheng Chang, Yen-Ju Pan, Mohamad Nor Azra, Li-Li Chen, Te-Hua Hsu

**Affiliations:** 1 Department of Aquaculture, National Taiwan Ocean University, Keelung 20224, Taiwan; booshhoh@gmail.com (C.-H.M.); corry0826@gmail.com (Y.-C.C.); panyj@mail.ntou.edu.tw (Y.-J.P.); 2 Center of Excellence for the Oceans, National Taiwan Ocean University, Keelung 20224, Taiwan; abcm1042@hotmail.com (P.-Y.H.); joechen@ntou.edu.tw (L.-L.C.); 3 Institute of Marine Biology, National Taiwan Ocean University, Keelung 20224, Taiwan; 4 Higher Institution Centre of Excellence (HICoE), Institute of Tropical Aquaculture and Fisheries (AKUATROP), Universiti Malaysia Terengganu, Kuala Nerus 21030, Terengganu, Malaysia; azramn@umt.edu.my

**Keywords:** seaweed, cannibalism, aquaculture, hatchery, polyculture, agonistic behavior

## Abstract

**Simple Summary:**

Fighting with each other is a major problem in lobster and crab aquaculture. Reducing the fighting behavior of lobsters and crabs can improve survival during the culturing process. Juvenile lobsters and crabs were both cultured under different shelters (seaweed and cotton filter) and live prey conditions. Groups with shelter and co-culturing with live prey showed a better survival rate for both juvenile lobsters and crabs. Although providing shelter is currently the main method for reducing agonistic behavior, it must be continually altered as the lobsters and crabs grow. Live prey can grow and attract lobsters and crabs to hunt them, and live prey can be supplemented at any time. They can also be used as an additional source of income during the harvest season.

**Abstract:**

Cannibalism is a major problem in lobster and crab aquaculture. Reducing the aggressive characteristics of lobsters and crabs can improve survival during the culturing process. In this study, juvenile scalloped spiny lobsters (*Panulirus homarus*) and crucifix crabs (*Charybdis feriatus*) were both cultured under different shelter and live prey conditions. Groups with shelter (seaweed and cotton filter) showed a better survival rate than the control group (no shelter; *p* < 0.05) for both *Pa. homarus* and *Char. feriatus*. Co-culturing with live prey (*Litopenaeus vannamei*) significantly benefited the juveniles of *Pa. homarus* and visibly increased the survival of juvenile *Char. feriatus*. Although providing shelter is currently the main method for reducing agonistic behavior, it must be continually altered as the lobsters and crabs grow. Live prey can grow and attract lobsters and crabs to hunt them, and live prey can be supplemented at any time. They can also be used as an additional source of income during the harvest season.

## 1. Introduction

Crustaceans are an important fishery resource. In the inland and offshore fishery industries, crustacean aquaculture production accounted for approximately 9% of total world aquaculture from 1990 to 2020; it was approximately 9.4 million tons in 2018 [1]. At present, the main cultured species include whiteleg shrimp (*Litopenaeus vannamei*), red swamp crayfish (*Procambarus clarkii*), Chinese mitten crab (*Eriocheir sinensis*), giant tiger prawn (*Penaeus monodon*), oriental river shrimp (*Macrobrachium nipponense*), and giant freshwater prawn (*M. rosenbergii*) [1].

Despite aquaculture technology developing significantly during the past 20 years, only a few crustacean species have been produced commercially. Most species of crustaceans such as spiny lobster and marine crabs are still wild caught. With the high demand for human consumption, the problem of overfishing needs to be solved urgently [1]. Artificial breeding technology is strongly needed for reducing the pressure on the crustacean fishing industry, for resource restoration, and for stock enhancement.

Spiny lobsters are one of the most important and highly valued catches in the world, and their overfishing has become a serious problem. Between 2017 and 2019, the import and export volumes of lobsters in various major economies were generally stable, but the price fluctuations per unit were dramatic, indicating the instability of its fishery resources [2]. The artificial breeding and aquaculture technology of spiny lobsters are a rising concern. However, due to spiny lobsters’ long planktonic stage (6–12 months), high water quality requirements, and unknown nutritional requirements, the survival rate of seedlings is extremely low [3,4,5,6,7,8,9]. Currently, commercial aquaculture of spiny lobsters mainly involves the use of postlarvae (or pueruli) and juveniles from wild catches [8,9].

The crucifix crab, *Charybdis feriatus*, is characterized by a special surface pattern. It has a red body with dark brown patches and a cross-shaped pattern on the back. They have good economic value in East Asian countries and are considered a candidate species for aquaculture in the Indo-Pacific region [10]. Although some research has focused on its on artificial breeding, no available commercialization has been reported [11]. Furthermore, the price of crabs is demonstrating an upward trend and the problem of overfishing is emerging gradually [2].

In addition to the difficulties of breeding in the zooplankton stages, the juvenile period for crabs and spiny lobsters also results in high mortality due to cannibalism. Spiny lobsters and crabs are more aggressive than other crustacean species. The post-larval (pueruli of spiny lobsters and megalopas of crabs) and juvenile culture stages usually occur in a high-density environment and the survival rate greatly declines due to agonistic behavior.

The choice of shelter, feeding density, and feeding frequency is critical to the survival rate of postlarvae and juveniles [12,13,14,15,16,17,18,19]. Several artificial material shelters, including cement, plastic plates, PVC tubes, and wood have been used [13,14,17,20]. Oniam et al. [20] examined different shelters for the juvenile stage of blue swimmer crabs (*Portunus armatus*) and found that the type of shelter affects survival significantly. Supriyono et al. [17] used shelters in different densities to the number of lobsters (*Panulirus homarus*) and discovered that when the number of shelters is greater than the number of lobsters, the survival rate is higher.

Although shelters are useful, feeding methods are also important. Liu et al. [21] used clams (*Ruditapes philippinarum*) as bait to observe the hunting behavior of a pair of *Po. trituberculatus* crabs and found that increased bait density effectively reduced the competition between crabs. Clams do not move frequently or quickly, but when the density increases, they can effectively attract the crabs’ attention and reduce the occurrence of fighting behavior. Huang et al. [22] mixed Chinese mitten crabs (*E. sinensis*) with snails and aquatic plants to achieve almost double the survival rate of the control group. Although the cost is a poor harvest of snails, it effectively retains the more valuable *E. sinensis*.

In this study, juvenile scalloped spiny lobsters (*Pa. homarus*) and crucifix crabs (*Char. feriatus*) were cultured under different shelter and live prey conditions. Improving the survival rate during the seedling period by using shelters and live prey will greatly increase the aquaculture benefits.

## 2. Materials and Methods

### 2.1. Experimental Setup

The experiment was conducted in Gongliao Aqua Center, New Taipei City, Taiwan. *Pa. homarus* pueruli were collected from the wild (from Vietnam) and reared in 2500 L round FRP tanks. They were raised for 2–3 days (postlarvae, aged 4–6 days; body length 31.71 ± 6.52 mm) (Table S1) for all experiments. Mature *Char. feriatus* were collected from Keelung, Taiwan. After spawning, zoea 1 were collected and transferred to a 200 t cement tank for breeding. The rearing process is shown in Table S2. Juvenile crabs were divided into two groups (based on size) after harvest: + body width, 21.13 ± 2.10 mm (C6–C7); − body width 15.89 ± 0.69 mm (C5–C6). In total, 300 crabs were harvested for direct experimentation (Table S3). In the experimental groups, the body size of juvenile crabs in the same group was within the same range. All experimental spaces consisted of a flow-through open system with a temperature of 23–25 °C and salinity of 30–33%. 

### 2.2. Palinurid Lobster Experiment

The experimental groups are shown in Table 1. The *Pa. homarus* shelter experiment used seaweed (*Chaetomorpha crassa*) and cotton filter as shelter. The shelter test used 100 *Pa. homarus* postlarvae in each group (with three repeats). The rearing spaces were cages with a length of 89 cm, width of 59 cm, and water height of 33 cm. The seaweed and the cotton filter occupied approximately two-thirds of the cage. The feed was small fish pieces. They were fed two times a day, at 10:00 a.m. and 6:00 p.m., with leftover feed and dead individuals removed before feeding. The experiment was conducted for 20 days. In the prey experiment, fresh clams (*R. philippinarum*), fresh shrimp, and live shrimp (*L. vannamei*) were used as prey. Each group consisted of 10 palinurid lobster postlarvae, and the rearing spaces were tanks with a length of 60 cm, width of 30 cm, and water height of 29.5 cm. The feed was divided into fresh clams and live shrimp (ML); fresh shrimp and live shrimp (SL); fresh clams, fresh shrimp, and live shrimp (MSL); fresh clams (M); fresh shrimp (S); and fresh clams and fresh shrimp (MS), with five repeats, each. They were fed two times a day, at 10:00 a.m. and 6:00 p.m., with leftover feed and dead individuals removed before feeding. The number of live shrimps was maintained at 10, and the body length was approximately equal to *Pa. homarus*. The experiment was conducted for 7 days.

### 2.3. Crucifix Crab Experiment

The experimental groups are shown in Table 1. The *Char. feriatus* shelter experiment used seaweed (*Chae. crassa*) and cotton filter as shelter. Before the shelter experiment, we tested the cannibalism of *Char. feriatus* in tanks (without shelters) and obtained low survival states (Tables S4 and S5) caused by the higher chance of encounter. The larger rearing space requirement was also checked before the shelter experiment (Figure S1), so the following experiments all used cages as a rearing space. The shelter test used 20 juvenile crabs in each group (including the control group) with three repeats each. In total, 180 juvenile crabs were used in the experiment for 22 days. The rearing spaces were cages with a length of 89 cm, width of 59 cm, and water height of 33 cm. The seaweed and the cotton filter occupied approximately two-thirds of the cage (Figure S2). The feed was small fish pieces. They were fed two times a day, at 10:00 a.m. and 6:00 p.m., with leftover feed and dead individuals removed before feeding. In the prey experiment, live shrimp (*L. vannamei*) and live clams (*R. philippinarum*) were used as the prey. Each prey group was divided into three densities: low density (3 crabs), middle density (5 crabs), and high density (10 crabs). The experiment was carried out for 22 days and a total of 72 juvenile crabs were used. The rearing spaces were cages with a length of 89 cm, width of 59 cm, and water height of 33 cm. The number of live shrimp was equal to and maintained at the number of juvenile crabs in the group. The length of live shrimp was divided into two sizes: 2–3 cm (live shrimp S) and ≥4 cm (live shrimp L). The number of live clams was fixed at 20. Dead prey was not removed or replaced during the experiment. They were fed two times a day, at 10:00 a.m. and 6:00 p.m., with leftover feed and dead individuals removed before feeding.

### 2.4. Statistical Analysis

In this study, we used a completely randomized design (CRD). Survival data were statistically analyzed using the analysis of variance (ANOVA) *F*-test, with a 95% confidence interval in Microsoft Excel 2019. If it was significantly different, Tukey’s test was performed to examine the differences between treatments.

## 3. Results

**Figure 1:**
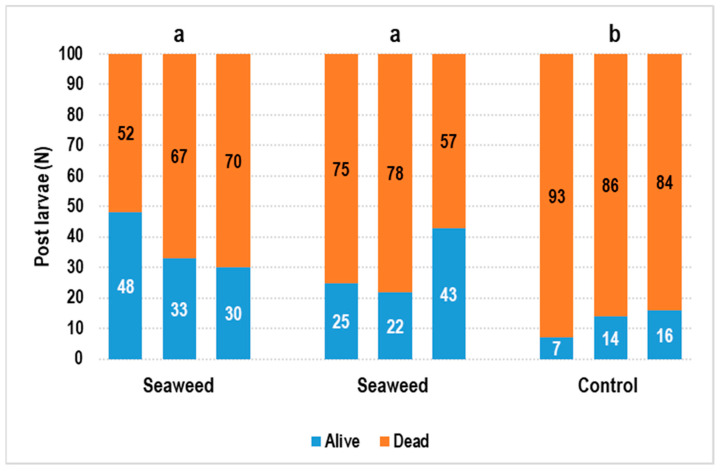
*Panulirus homarus* shelter experiment. In the shelter groups (seaweed and cotton filter), the survival rate was significantly higher than in the control group (^a,b^ *p <* 0.05).

In the *Pa. homarus* shelter experiment, we saw that the survival status of the shelter group was better than that of the control group, with significant differences (*p <* 0.05; Figure 1). However, final survival results showed high variation within groups. After 20 days, the maximum survival rate was only 48% and 43% and the minimum survival rate was 30% and 22% in the shelter groups (Figure 1). This problem was potentially related to density. The density in this experiment was approximately 190 ind/m^2^.

**Figure 2:**
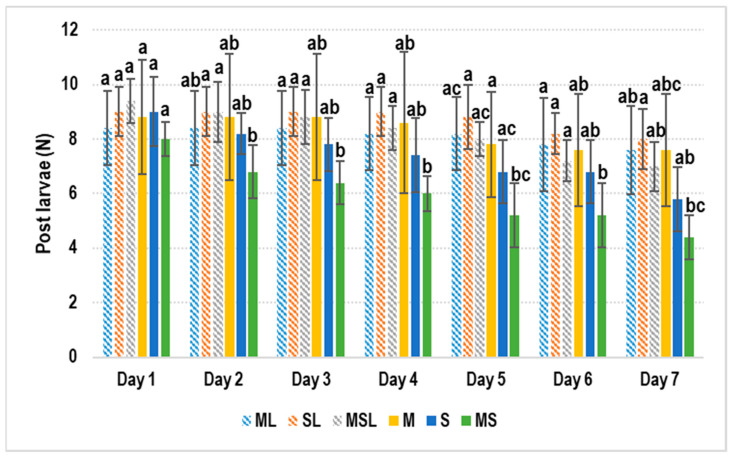
*Panulirus homarus* prey experiment. The results showed a significant difference on Day 2. There were no significant differences between the three groups using live shrimp but there were significant differences from the non-live shrimp group. ML: Fresh clams and live shrimp; SL: Fresh shrimp and live shrimp; MSL: Fresh clams, fresh shrimp, and live shrimp; M: Fresh clams; S: Fresh shrimp; MS: Fresh clams and fresh shrimp (^a,b,c^ *p <* 0.05).

In the prey experiment, all groups experienced residual fighting on Day 1 (density ≈ 56 ind/m^2^), and the groups containing live shrimp mostly performed well. This showed that the interference effect of the live prey was greater than the non-live prey, and the difference appears after 7 days (Figure 2).

**Figure 3:**
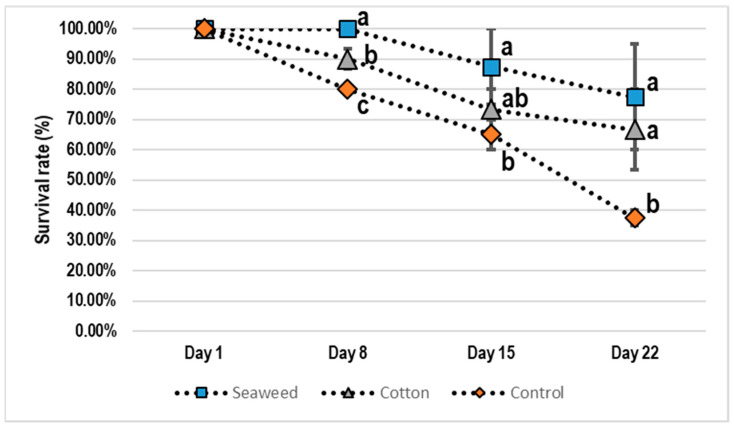
*Charybdis feriatus* shelter experiment. There was a significant difference at the beginning of the shelter experiment on Day 8, and then the survival rate of control gradually reduced. ■ seaweed; ▲ cotton filter; ◆ control (^a,b,c^ *p <* 0.05).

In the *Char. feriatus* shelter experiment, there was a significant difference at the beginning of the shelter experiment on Day 8. Over time, while survival rates in all treatments declined, survival of groups in the seaweed and cotton shelters was higher than that in the control group. The final survival results demonstrated a high variation within the groups. The survival rate of the seaweed groups was higher than that of the cotton filter groups (Figure 3). The experimental results indicated that shelters were positive for the survival of juvenile crabs (>60% survival).

**Figure 4:**
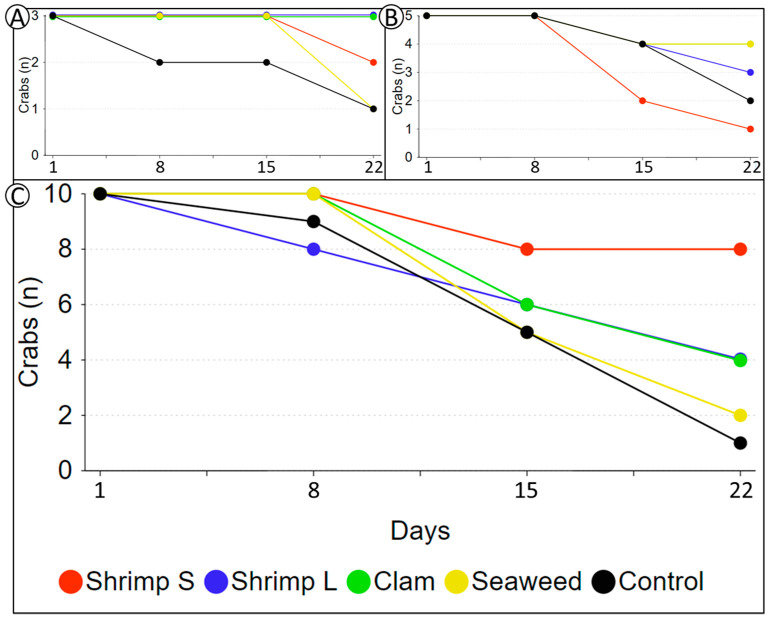
*Charybdis feriatus* prey experiment. All the prey groups showed higher survival than the control in the high-density groups: (**A**) low density (3 crabs); (**B**) middle density (5 crabs); (**C**) high density (10 crabs).

In the prey experiment, there was no statistically significant difference in the results of the experiment. The figures indicate that when the density of juvenile crabs increased, the efficiency of the two treatments using live shrimp was higher than that of the control (Figure 4). The survival rate, from high to low, was as follows: N = 3, Shrimp L = Clam > Shrimp S > Control; N = 5, Clam > Shrimp L > Control > Shrimp S; and N = 10, Shrimp S > Shrimp L = Clam > Control. From these results, it is clear that the survival rate of groups with live organisms as prey is generally higher (Figure 4). The use of live prey and the size of prey in the polyculture can increase the survival rate.

## 4. Discussion

Despite aquaculture technology developing significantly over the past 20 years, spiny lobster and marine crabs are still not produced commercially. Although some research has focused on the artificial breeding of *Charybdis feriatus*, no available culture method has been reported [11]. In this study, we aimed to produce the juvenile *Char. feriatus* and try to find a suitable culture method for the juvenile stage. Extreme cannibalism behavior was found in the juvenile stage (C5–C6). Whether the rearing density was high (group) or low (pair), cannibalism was significant (Tables S4 and S5). The use of shelters to prevent fighting is currently the main method of lobster and crab farming. However, shelter size, material, and appearance often affect the final survival rate [15,20]. When selecting a shelter, most studies used artificial material shelters, such as cement, plastic plates, PVC tubes, or wood [13,14,17,20]. Eggleston et al. [13] reported that *Pa. argus* will change its choice of shelter as it grows in size. However, it is inconvenient to change the shelter as lobsters are growing.

This study was the first to use seaweed (*Chae. crassa*) as a shelter for juvenile *Pa. homarus* and *Char. feriatus*. We found that *Chae. crassa* can provide a suitable shelter for juvenile lobsters and crabs. It is easy to change the density of the seaweed shelter for different sizes of lobsters and crabs. Seaweed grows easily and does not need to be replaced. However, if the seaweed grows too much, the shelter will take over the space, and *Pa. homarus* and *Char. feriatus* juveniles will no longer find a hiding spot. Using seaweed as shelter results in a better survival rate than using a cotton filter shelter for *Pa. homarus* and *Char. feriatus* (Figure 1 and Figure 3).

Seaweed, aquatic plant use in polyculture, or integrated multitrophic aquaculture (IMTA) systems, are common and have a positive effect on water quality. Huang et al. [22] reported a polyculture experiment involving *E. sinensis* in which aquatic plants were used for water quality control. Sumbing et al. [16] tested *Kappaphycus alvarezii* (red algae), *Pa. ornatus* (ornate spiny lobster), and *Holothuria scabra* (sea cucumber) for the IMTA system, and the survival rate of *P. ornatus* was as high as 90% during the 10-week experimental period. Notably, Sumbing et al. [16] did not culture *Kappaphycus alvarezii* with *P. ornatus* in the same tank; *K. alvarezii* was used for water quality control in the system. Gao et al. [23] reported that *Chae. crassa* has the potential to serve as a biological filter for the reduction of eutrophication. It shows a high capacity for growth and nitrogen accumulation and a greater physiological tolerance of low salinity during periods of elevated temperature.

Some studies have shown that seaweed may provide additional feed sources for lobsters and crabs. Joll [24] reported that *Pa. cygnus* ate red algae. Castañeda-Fernández-de-Lara et al. [25] and Mashaii et al. [26] found that *Pa. homarus* could eat some algae. Baeza et al. [18] tested the chemical sensing of *P. argus* postlarvae and found that lobsters are attracted to algae. However, during our experiment, we did not find that juvenile *Pa. homarus* and *Char. feriatus* ate *Chae. crassa*. We tested a water extract of *Chae. crassa* and found that it could stabilize lobsters and crabs (data not shown). This may provide another reason for the higher survival rate compared to the cotton filters; *Chae. crassa* were not eaten by those crustaceans (based on this study), and the algae were not consumed during rearing.

Despite the important role that shelter plays in lobster and crab culturing, we focused more on the positive effects of live prey. Feeding live prey is easy for juvenile *Pa. homarus* and *Char. feriatus*. It can significantly decrease fighting behavior and provide sufficient supplemental nutrition. We saw a higher survival rate for *Pa. homarus* using live shrimp than the non-living prey groups (Figure 2). In the *Char. feriatus* prey experiment, high-density groups (10 crabs) demonstrated notably positive results when using live shrimp as prey. Although there was no significant difference in our study, we hypothesize that selecting live prey of a suitable size attracts the attention of crabs, and hunting this prey effectively reduces fighting due to fewer interactions between crabs. Small live shrimp achieved better results than large live shrimp in high-density groups, supporting this hypothesis regarding prey size (Figure 4).

Most lobster and crab culture systems experience critical fighting problems. Although cage aquaculture is widely used for lobsters, it is not suitable for juvenile *Pa. homarus* or *Char. feriatus* [27]. Using a single cage or box for crabs has been employed, but it is not economic for juvenile *Pa. homarus* or *Char. feriatus*; it is more suitable for adult crabs. Using plastic shelters has its limitations for different size ranges, but seaweed is relatively flexible (Figure S2). Using live prey is expensive, but a reasonable alternative to improve the survival of the juvenile *Pa. homarus* and *Char. feriatus*. Moreover, live clam (*R. philippinarum*) and shrimp (*L. vannamei*) are cheap feed for juvenile *Pa. homarus* and *Char. feriatus* (Figure 2 and Figure 4). Live prey such as *R. philippinarum* and *L. vannamei*, which are the main cultured species in the world, are easy to obtain. Polyculture or IMTA of juvenile *Pa. homarus* and *Char. feriatus* using *R. philippinarum* and *L. vannamei* is viable, and the availability of shrimp can increase the lobster and crab harvest. Further development of live prey for polyculture is thus warranted; we have not further shown the effectiveness of seaweed and live prey at the same time. This IMTA method could be a possible direction toward improving the survival of lobsters and crabs in their juvenile period.

## 5. Conclusions

In this study, we found that groups with shelter and co-culturing with live prey showed better survival rates for both juvenile lobsters and crabs. Although providing shelter is currently the main method for reducing agonistic behavior, it must be continually altered as the lobsters and crabs grow. Live prey can grow and attract lobsters and crabs to hunt them. Moreover, live prey can be supplemented at any time and can be used as an additional source of income during the harvest season. The IMTA method (seaweed, clam, and shrimp) could be a possible direction toward improving the survival of lobsters and crabs in their juvenile period.

## Figures and Tables

**Table 1:** Experimental groups.

Organism	Subject	Treatment
*Panulirus homarus*	Shelter	Seaweed
		Cotton filter
	Prey	ML: Fresh clam and live shrimp
		SL: Fresh shrimp and live shrimp
		MSL: Fresh clam, fresh shrimp, and live shrimp
		M: Fresh clam
		S: Fresh shrimp
		MS: Fresh clam and fresh shrimp
*Charybdis feriatus*	Shelter	Seaweed
		Cotton filter
	Prey	Live shrimp S
		Live shrimp L
		clam

## Data Availability

Not applicable.

## Supplementary Materials

The following are available online at https://www.mdpi.com/2076-2615/11/2/370/s1, Figure S1: Space requirement test for *Charybdis feriatus*. Each space was rearing one crab individually with 10 replicates. The tank group got 100% survival during the test, next was the Bottle group 50% and the PVC plate was 10%. Tank: length 60 cm, width 41.5 cm and height 9.5 cm; Bottle: circular bottom diameter 7.5 cm, 400-mL; PVC plate: circular bottom diameter 7.5 cm, 100 mL. Size of juvenile crabs: average weight: 1.05 ± 0.50 g; average carapace width: 16.10 ± 2.62 mm, Figure S2: Seaweed (*Chaetomorpha crassa*) for the *Charybdis feriatus* shelter experiment, Table S1: *Panulirus homarus* pueruli to postlarvae (PL, N = 5), Table S2: *Charybdis feriatus* zoea and megalopa rearing process, Table S3: Juvenile to mature *Charybdis feriatus* (N = 5), Table S4: Pair cannibalism test for *Charybdis feriatus*. Juvenile crabs (pair) were rearing in tanks (the bottom area is 1800 cm^2^), Table S5: Group cannibalism test for *Charybdis feriatus*. Ten juvenile crabs (group) were rearing in tanks (the bottom area is 1800 cm^2^).
