# Supplementary material for: Improving Survival of Juvenile Scalloped Spiny Lobster (Panulirus homarus) and Crucifix Crab (Charybdis feriatus) Using Shelter and Live Prey
Source: Animals (Basel). 2021 Feb 2;11(2):370. doi: 10.3390/ani11020370 (PMC7913089; doi:10.3390/ani11020370)
Supplement: Supplementary file 1 [file animals-11-00370-s001.pdf]

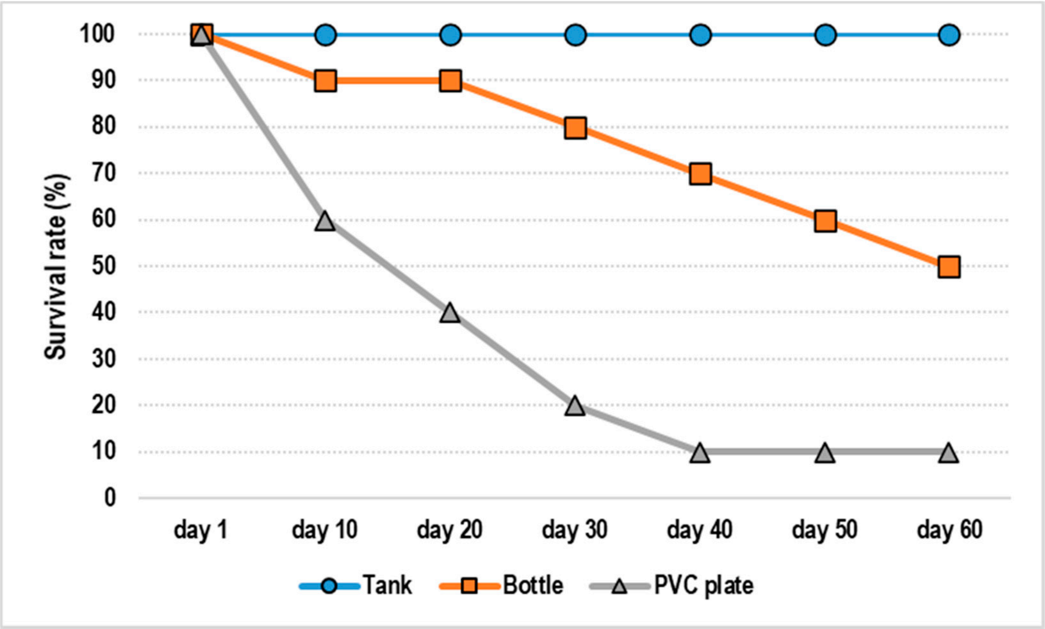

Figure S1. Space requirement test for *Charybdis feriatus*. Each space was rearing one crab individually with 10 replicates. The tank group got 100% survival during the test, next was the Bottle group 50% and the PVC plate was 10%. Tank: length 60cm, width 41.5cm and height 9.5cm; Bottle: circular bottom diameter 7.5cm, 400-ml; PVC plate: circular bottom diameter 7.5cm, 100ml. Size of juvenile crabs: average weight: 1.05 ± 0.50 g; average carapace width: 16.10 ± 2.62 mm.

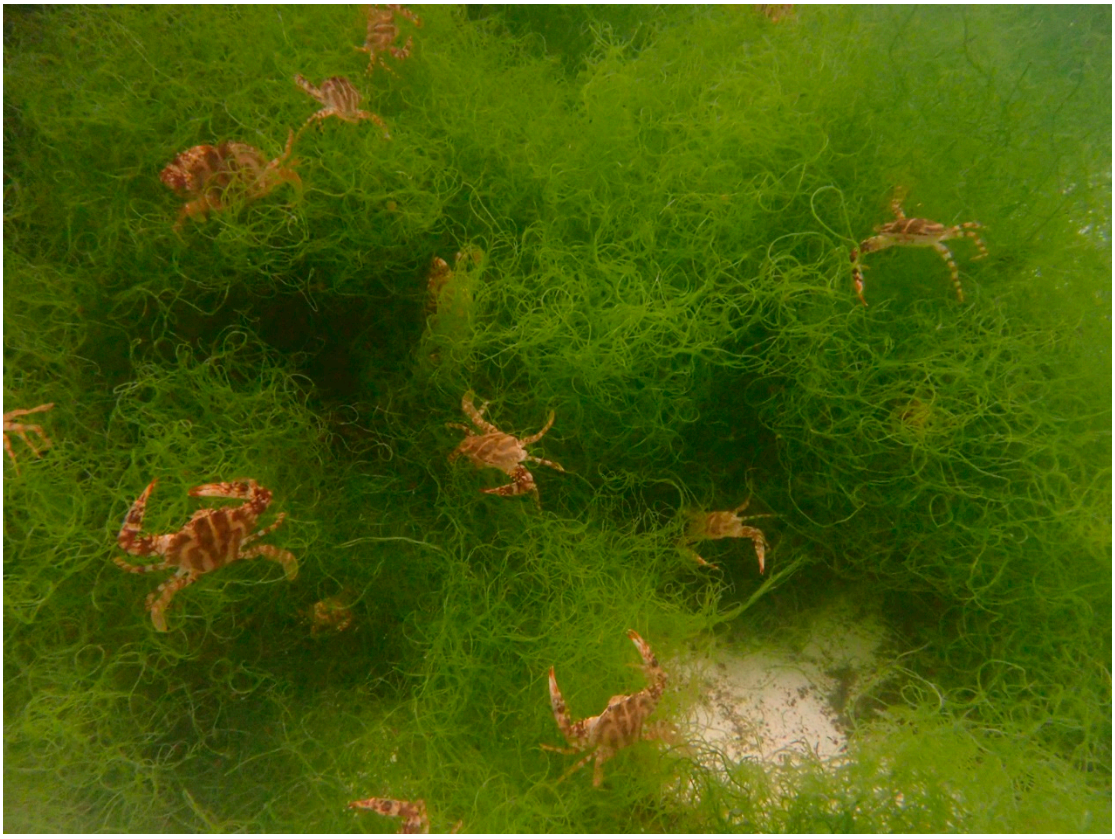

Figure S2. Seaweed (*Chaetomorpha crassa*) for the *Charybdis feriatus* shelter experiment.

Table S1. *Panulirus homarus* pueruli to postlarvae (PL, N = 5)

Stage	Carapace width (cm)	Growth rate%
pueruli	1.23	
PL 1	1.4	14%
PL 2	1.75	25%
PL 3	2.2	26%
PL 4	2.5	14%
PL 5	3	20%
PL 6	3.78	26%
PL 7	5.05	35%

Table S2. *Charybdis feriatus* zoea and megalopa rearing process

Crab larval stage	Prey	During days
Zoea 1	Rotifer (density of 0.1-0.5/ml)	3-5 days
Zoea 2	Rotifer (density of 0.5-1/ml)	3-4 days
Zoea 3	Rotifer (density of 0.5-1/ml)<br>Copepod (density of 0.5-1/l)	3-5 days
Zoea 4	Rotifer (density of 0.1-0.5/ml)<br>Copepod (density of 0.5-1/l)	3-5 days
Zoea 5	Copepod (density of 0.5-1/l)<br>*Artemia* nauplii (density of 0.5-1/l)	3-5 days
Zoea 6	Copepod (density of 0.5-1/l)<br>*Artemia* nauplii (density of 0.5-1/l)	5-6 days
Megalopa	Copepod (density of 0.5-1/l)<br>*Artemia* nauplii (density of 0.5-1/l)<br>Fresh mysis shrimp (wet weight 100g)	3-4 days

Table S3. Juvenile to mature *Charybdis feriatus* (N = 5)

Stage	Carapace width (cm)	Growth rate%
C1	0.3	
C2	0.5	67%
C3	0.7	40%
C4	1	43%
C5	1.3	30%
C6	1.6	23%
C7	2.4	50%
C8	3.2	33%
C9	4.2	31%
C10	6.4	52%
C11	7.3	14%
C12	8.5	16%

Table S4. Pair cannibalism test for *Charybdis feriatus*. Juvenile crabs (pair) were rearing in tanks (the bottom area is 1800 cm^2).

Repeats	Before cannibalism happened (days)
Pair 1	12
Pair 2	0
Pair 3	5
Pair 4	2
Average days	4.75 ± 4.548

Size of juvenile crabs: average weight: 2.25 ± 0.60 g; average carapace width: 23.63 ± 1.93 mm.

Table S5. Group cannibalism test for *Charybdis feriatus*. Ten juvenile crabs (group) were rearing in tanks (the bottom area is 1800 cm^2).

Repeats	Day 1	Day 7	Survival rate (%)
1	10	4	40 %
2	10	3	30 %
3	10	5	50 %
Average survive (N)	10 ± 0.0	4 ± 0.8	40 ± 8.2 %

Size of juvenile crabs: aver weight: 1.09 ± 0.62 g; average carapace width: 17.30 ± 3.52 mm.
